# A Summary of the United States Food and Drug Administrations’ Food Safety Program for Imported Seafood; One Country’s Approach

**DOI:** 10.3390/foods5020031

**Published:** 2016-04-29

**Authors:** Brett Koonse

**Affiliations:** U.S. Food and Drug Administration, Center for Food Safety and Applied Nutrition, College Park, MD 20740, USA; brett.koonse@fda.hhs.gov; Tel.: +1-240-402-1401

**Keywords:** seafood safety, imports food safety, FDA seafood

## Abstract

It is well known that the vast majority of seafood is captured or farmed in emerging countries and exported to developed countries. This has resulted in seafood being the number one traded food commodity in the world. Food safety is essential to this trade. Exporting countries should understand the regulatory food safety programs of the countries they ship to in order to comply with their applicable laws and regulations to avoid violations and disruptions in trade. The United States (U.S.) imports more seafood than any individual country in the world but the European Union (E.U.) countries, as a block, import significantly more. Each importing country has its own programs and systems in place to ensure the safety of imported seafood. However, most countries that export seafood have regulatory programs in place that comply with the import requirements of the E.U. The purpose of this paper is to describe the United States Food and Drug Administration’s (USFDA) imported seafood safety program. The primary audience for the information is foreign government regulators, seafood exporters, and U.S. importers. It can also give consumers confidence that f U.S. seafood is safe no matter which country it originates from.

## 1. Introduction

All major seafood receiving countries have their own requirements to ensure the safety of the seafood they import. This presents a challenge to exporting countries because they have to comply with the requirements of each country to which they export. The collective countries of the European Union make up the largest importer of seafood in the world. The European Commission’s Directorate-General for Health and Consumers (SANCO) is responsible for seafood safety in the European Union [1]. Because the E.U. collectively is the largest importer of seafood and has historically worked closely with the exporting country’s seafood safety government authority, most major seafood exporting countries understand the SANCO program and have export programs in place that comply with SANCO’s requirements.

SANCO has a diverse food safety program that includes a mandatory “recognition” of the official governmental competent authority within the exporting country, various strategies for testing seafood products offered for entry into the member states, training and technical assistance to countries, and other programs. A particular focus of the SANCO program is the requirement that the competent authority develop and maintain a “list” of establishments (e.g., processors, cold storage facilities, *etc.*) that conform to SANCO requirements or directives. There are also specific requirements for raw material sites, such as the structure of harvesting vessels, landing locations, and aquaculture operations. Another requirement is that shipments into the E.U. must be accompanied by the appropriate health certificates. Audits of the competent authority are conducted to measure their conformance with these and other SANCO requirements.

The lead authority for seafood safety in the United States is the U.S. Food and Drug Administration (USFDA). While the USFDA has a number of requirements that are similar to SANCO’s, there are significant differences. Some of the differences are: the USFDA does not identify or recognize a country “competent authority”; does not require a country to maintain a list of processors that conform to USFDA requirements; does not require a processor or exporter of seafood to obtain or include health certificates with each shipment of product to the U.S.; and does not conduct audits of the competent authority in order to measure conformance with USFDA requirements. In addition, while the USFDA has a variety of sanitation and adulteration requirements for all food (e.g., the use of potable water and ice, the requirement that all drugs used on aquaculture farms be approved by the USFDA), there are no specific food safety regulatory programs for aquaculture operations, landing sites, or harvesting vessels. Therefore, because most of the exporting countries are more informed about the E.U. SANCO program and there are significant differences between SANCO and the USFDA, it is important to inform the foreign seafood exporting industry, governments, and others about the USFDA seafood import requirements and programs.

**Figure 1:**
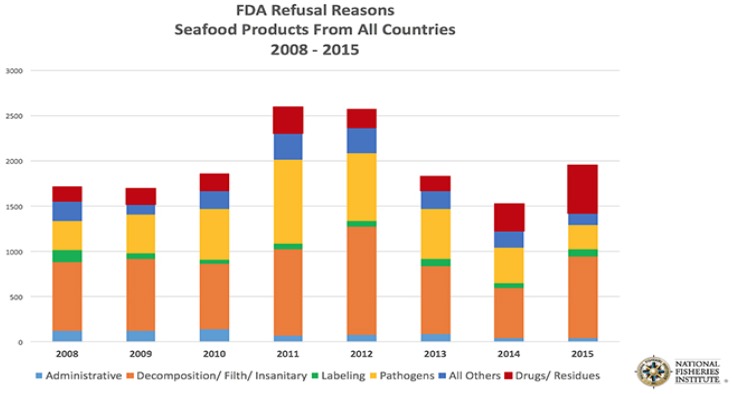
United States Food and Drug Administration (USFDA) Seafood Product Refusals.

If a country exporting seafood to the U.S. is not aware of the requirements for imported seafood and fails to comply with U.S. laws and regulations their products may not be allowed entry into the U.S. The USFDA refused nearly 2000 shipments of imported seafood in 2015, one of the highest totals in recent years (Figure 1) [2]. More than one-fourth of those refusals were because illegal substances were detected, frequently unapproved veterinary drugs administered to farmed fish and shellfish. Shipments that contained the unapproved drug residues were denied entry into the U.S. and were required to be shipped back to the country of origin. This high violation rate may illustrate a need to better inform the seafood exporting industry about the USFDA seafood safety requirements, including the requirement that only USFDA approved drugs can be used to treat aquacultured animals intended to be exported as seafood to the U.S.

## 2. Discussion

### 2.1. Overview of the USFDA Seafood Import Food Safety Program

Seafood is one of the most highly-traded commodities in the world with the vast majority of available seafood in the U.S. is imported. The Department of Health and Human Services’ (HHS) Food and Drug Administration (USFDA) is the lead agency charged with ensuring the safety of fish and fishery products entering the United States. (The U.S. Department of Commerce’s National Marine Fisheries Service, the U.S. Department of Agriculture’s Food Safety Inspection Service, and individual states also have roles in ensuring the safety of seafood imported into the U.S.; however, they are not discussed in this paper.) The USFDA recognizes and understands that success in ensuring that the seafood in the U.S. market is safe depends on reaching beyond U.S. borders and engaging with its government regulatory counterparts in other nations, as well as with the seafood industry and regional and international organizations, to encourage the implementation of science-based standards to ensure the safety of seafood before it reaches the U.S.

The USFDA operates a mandatory safety program for all fish and fishery products under the provisions of the Federal Food, Drug and Cosmetic (FD&C) Act, the Public Health Service Act, and related regulations. The USFDA program is multi-faceted and includes the following:

- Mandatory Hazard Analysis Critical Control Point (HACCP) evaluations and HACCP plans if necessary;
- Foreign Inspections;
- Domestic Importer Inspections;
- Global Presence;
- Seafood Import Surveillance and Enforcement;
- Foreign Country Assessments;
- Food Safety Modernization Act requirements.

### 2.2. USFDA’s Mandatory Hazard Analysis and Critical Control Point (HACCP) Program

USFDA’s multifaceted and risk-informed seafood safety program relies on various measures of compliance with its seafood HACCP regulation. This is a management system in which food safety is addressed through the analysis and preventive control of biological, chemical, and physical hazards from raw material production, procurement, handling, manufacturing, and distribution to the final point of sale of the finished product.

The cornerstone of the HACCP program is the USFDA’s Fish and Fisheries Products Hazards and Controls Guidance document, an extensive compilation of the most up-to-date science and policy on the hazards that affect fish and fishery products and effective controls to prevent their occurrence. The fourth edition of this guidance document, which has become the foundation of fish and fishery product regulatory programs around the world, is now available.

Under the seafood HACCP regulation, HACCP controls are required for both domestic and foreign processors of fish and fishery products intended for the U.S. market. The regulation also requires that U.S. importers take certain steps to verify that their foreign suppliers meet the requirements of the regulation. USFDA uses a variety of measures to enforce processors’ compliance with seafood HACCP, including inspections of foreign processing facilities, use of a screening system to sample imported products, domestic surveillance sampling of imported products, inspections of seafood importers, , foreign country program assessments, and information from our foreign partners and USFDA foreign office posts.

### 2.3. Foreign Inspections

Each year since 1999 the USFDA has inspected a limited number of seafood processors that export to the U.S. In recent years, the USFDA has significantly increased the number of inspections it conducts of foreign food manufacturers, including seafood manufacturers. For example, in fiscal year 2008 the USFDA conducted 303 inspections of foreign food facilities, of which 95 were seafood processors. In fiscal year 2014 the USFDA conducted 1336 foreign food facility inspections of which 303 were seafood processors. Every seafood processor whose product is intended for the U.S. market is required to have and implement a written HACCP plan whenever a hazard analysis reveals at least one food safety hazard that is reasonably likely to occur. The HACCP inspection approach, for the purpose of verifying compliance with the seafood HACCP regulation, is used by USFDA during domestic and foreign inspections of seafood processors to focus its attention on the parts of the process that are most likely to affect the safety of the product. In contrast to historical methods of evaluating processing practices on the day of the inspection, the HACCP approach allows USFDA to evaluate processors’ overall implementation of their HACCP systems over a period of time. This is done by accessing the firms’ HACCP plans and determining if the pertinent hazards have been identified. If they have been identified the investigator checks to see if appropriate preventive controls are in place, and verifies whether or not the firm has monitoring records and corrective action records on file. In this model, it is the seafood industry’s responsibility to develop and implement HACCP controls and the regulatory agency’s role to ensure that the industry complies.

It is important to note that HACCP compliance is only one element of a USFDA inspection. The seafood HACCP regulation is complemented by other regulations, including the Current Good Manufacturing Practice regulation, which provides the basis for determining whether the products have been processed under sanitary conditions, and the Thermally Processed Low-Acid Foods Packaged in Hermetically Sealed Containers and Acidified Food regulation, which specifically addresses the *Clostridium botulinum* hazard in these products. Together, these regulations provide the regulatory food safety controls to which a processor of fish or fishery product is subject.

The frequency of inspection for foreign firm is determined by risk-based product priorities as well as other country-specific factors, including the volume of seafood exported to the U.S., the history of violations associated with the products originating from the country, the outcome of previous inspections conducted of the seafood processors, the outcome of importer inspections, the credibility of information raising safety concerns with a foreign establishment’s or country’s exports, and the use of a new technology or process by processors that might raise food safety concerns. For example, countries or individual processors that process and export known high priority products, such as vacuum packed raw fish, ready-to-eat fishery products, scombrotoxin-forming fish and aquaculture seafood, are routinely targeted for inspection.

Although inspection frequency is based primarily on risk-based product and country priorities, the USFDA may adjust the frequency if a particular country, region or specific establishment is connected with violative surveillance samples or is associated with an illness outbreak or other event that causes the USFDA to be concerned about the safety of the seafood (e.g., a natural disaster such as a hurricane, earthquake, tsunami, or an aquaculture disease outbreak which may result in the use of illegal or unapproved antibiotics).

The regulatory options available for the USFDA with respect to foreign processors that do not comply with USFDA regulations include placing the affected products on Import Alert for detention without physical examination (DWPE). This means the product is subject to detention or refused admission into the U.S. until it is demonstrated to be in compliance.

### 2.4. Domestic Seafood Importer Inspections

It is the importer’s responsibility to offer for entry into the U.S. products that are fully compliant with all applicable U.S. laws. Under the seafood HACCP regulation, HACCP controls are required for both domestic and foreign processors of fish and fishery products. Additionally, the regulation requires that U.S. importers take certain steps to verify that their foreign suppliers meet the requirements of the regulation.

The importer must meet its obligation by having and implementing written verification procedures for ensuring that the fish and fishery products offered for entry into the U.S. were processed in accordance with the requirements of the regulation. Some verification steps taken by importers include: maintaining a current copy of the foreign processor’s HACCP plan along with the processor’s written guarantee of compliance with the seafood HACCP regulation, inspecting the foreign processor’s facilities to ensure compliance with the seafood HACCP regulation, and obtaining continuing or lot-by-lot certifications from an appropriate foreign government inspection authority certifying that the products were processed in compliance with the seafood HACCP regulation.

USFDA conducts inspections of domestic seafood importers to verify compliance with these seafood HACCP requirements. Similar to foreign processor inspections, USFDA prioritizes importer inspections. Importers who seek to bring products with a history of violations into the U.S. or who import from a processor that has a history of compliance deficiencies, may be inspected at a greater frequency than importers dealing with products and processors who do not have a history of violations.

### 2.5. Seafood Import Surveillance and Enforcement

**Figure 2:**
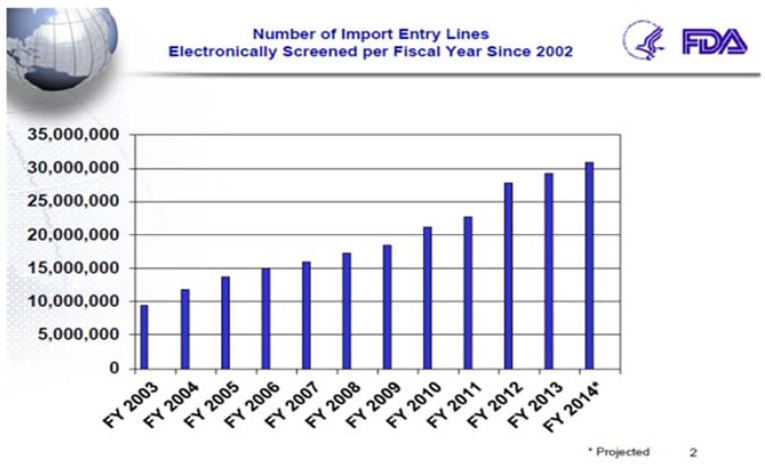
Number of USFDA Import Entry Lines.

Between 2002 and 2010 overall U.S. food imports, as measured by the number of “lines” of imported food, almost doubled from 4.4 million to 8.6 million import lines (Figure 2). This trend is expected to continue and the U.S. public is likely to consume even more imported food in coming years.

USFDA electronically screens all food offered for entry into the U.S., and a subset of those are physically inspected at varying rates, depending on the potential risk associated with them. To accomplish this USFDA has implemented an automated screening tool, the Predictive Risk-based Evaluation for Dynamic Import Compliance Targeting (PREDICT) system, which significantly improves the screening of imported food. PREDICT uses automated data mining and pattern discovery to identify data anomalies with regard to import and compliance history of a firm and/or a specific product, such as the facility inspection history; results of previous field exams, sample analyses, and facility inspections; and types of products that the firm offers for entry into U.S. commerce. For example, if a firm historically imports fresh seafood and suddenly imports canned seafood, this information is detected by PREDICT and may trigger a decision by the Agency to conduct an examination of the new type of imported product.

Once an entry is selected, USFDA will examine or analyze it for microbiological contamination, parasites, decomposition and histamine, chemical contaminants (e.g., pesticides, dioxin, *etc.*), food and color additives, filth, mold, foreign objects, unapproved new animal (aquaculture) drugs, packaging, and labeling. The type of examination and analysis depends on the product and the types of problems that have been associated with it in the past. USFDA may increase its sampling of certain products if current surveillance sampling detects a pattern of violative products from its source (country, foreign processor, or shipper). Increased sampling, beyond that which is part of the annual work plan, may be initiated via a sampling assignment or an Import Bulletin. In FY 2014, USFDA processed approximately 938,000 entries of imported seafood, performed nearly 26,000 physical examinations of seafood imports, and collected over 5600 samples of domestic and imported seafood for analysis at USFDA field laboratories [3].

When USFDA detects adulterated seafood or observes that an importer or foreign processor has failed to implement adequate safety (HACCP) controls, those subsequent shipments may be placed on DWPE when introduced for entry into the U.S. USFDA uses a system of publicly accessible Import Alerts to provide information and instructions to its field import review staff on how to process particular entries, including products that are subject to DWPE.

Products from firms listed on an Import Alert and subject to DWPE may be refused entry unless the owner or consignee of the goods provides evidence to USFDA that the seafood is not violative. In cases where USFDA determines that a particular problem is widespread in a country or region, the USFDA may place all of a particular type of product from that country or region on an Import Alert for DWPE. The number of Import Alerts varies but FDA currently has 38 Import Alerts relating to import seafood products.

USFDA's import surveillance system works in conjunction with USFDA's enforcement of the Seafood HACCP Regulation. For example, the finding of non-compliant product during import surveillance can result in USFDA scheduling an inspection of the importer or foreign processor.

### 2.6. USFDA Global Presence

Today, with 300,000 foreign facilities from more than 150 countries exporting USFDA-regulated products to the United States, USFDA works beyond U.S. borders to ensure that products coming into the United States are safe and effective. This includes working with governments, industry, and academia in foreign countries, as well as with multilateral organizations [4].

This is an important strategy because USFDA faces ever-greater challenges in determining whether a product has been properly manufactured, distributed, and stored, and in determining who has handled the product. The manufacture of a single product can involve multiple parties from different countries that are engaged at various steps throughout the process. Along the way, there are opportunities for the product to be improperly formulated or packaged, contaminated, diverted, counterfeited or adulterated.

**Figure 3:**
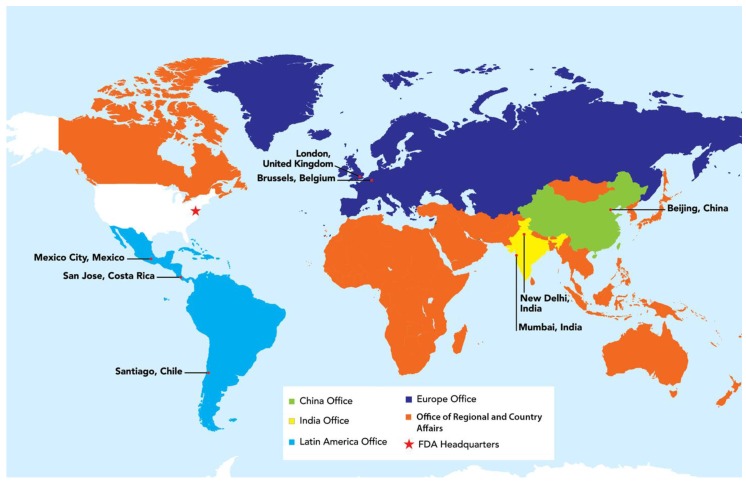
USFDA Foreign Office Locations.

USFDA utilizes diverse approaches to address the complex issues posed by globalization. In 2008, USFDA began establishing foreign offices, posting staff in strategic locations around the world, including China, Europe, India, and Latin America (Figure 3). USFDA overseas offices work closely with foreign governments, industry, and other stakeholders to enable USFDA to more effectively protect U.S. consumers.

These offices help USFDA:

- facilitate collaboration with foreign regulators and other stake holders;
- coordinate food safety issues with the competent authorities in the countries/regions in which they are located, such as response to food-borne illnesses traced to imported product;
- educate foreign industry about USFDA requirements;
- provide technical assistance to foreign competent authorities and industries; and
- inspect foreign manufacturers by placing inspectional staff permanently within the high priority countries/regions.

### 2.7. Foreign Country Assessments of Aquaculture Food Safety

The majority of seafood imported into the U.S. is from aquaculture operations. For this reason, understanding and ensuring the safety of seafood and aquaculture products is an essential component of USFDA’s efforts.

Foreign country assessments are systems reviews that offer USFDA a broad view of the ability of the country’s industry and regulatory infrastructure to control aquaculture drugs. These assessments allow USFDA to become familiar with the controls that a country’s competent authority implements over the distribution, availability, and use of animal drugs. USFDA uses country assessments to help determine the risk of unapproved drug residues in the aquaculture products a country may ship to the United States. Some of these unapproved drugs are suspected carcinogens; others present concerns for the development of antibiotic resistance in human bacterial pathogens.

This assessment program helps USFDA direct its resources more effectively and efficiently and allows USFDA to work directly with countries to resolve drug residues problems. The country assessment program also helps USFDA direct its foreign inspection and border surveillance resources more effectively and efficiently and allows USFDA to work directly with countries to resolve drug residue problems.

USFDA uses information from country assessments to:

- better target (*i.e.*, increase or decrease) surveillance sampling of imported aquaculture products;
- inform its decisions on what new analytical methods it needs to develop and what drugs or chemicals it should target for surveillance sampling;
- inform its planning of foreign seafood HACCP inspections;
- provide additional evidence for potential regulatory actions, such as an import alert;
- improve collaboration with foreign government and industry contacts to achieve better compliance with USFDA’s regulatory requirements; and
- better understand the causes for significant changes in a country’s drug residue problems, such as a sudden spike in noncompliant samples.

Some of the countries whose aquaculture food safety systems FDA has assessed include:

- China
- India
- Malaysia
- Indonesia
- Chile
- The Philippines
- Vietnam

### 2.8. Food Safety Modernization Act

The USFDA Food Safety Modernization Act (FSMA), the most sweeping reform of U.S. food safety laws in more than 70 years, was signed into law by President Obama on 4 January 2011. It aims to ensure the U.S. food supply is safe by shifting the focus from responding to contamination to preventing it. FSMA also gives USFDA new tools and authorities to help make certain imported foods meet the same safety standards as foods produced in the U.S. [5].

Seafood is specifically exempt from some of the new FSMA requirements. For example, the FSMA Foreign Supplier Verification Program (FSVP) for Importers of Food for Humans and Animals. This rule will require that importers of food perform certain risk-based activities to verify that food imported into the United States has been produced in a manner that meets applicable U.S. safety standards. Although importers of fish, and fishery products are exempt from this requirement, they are still subject to the seafood HACCP regulation which requires importers to have and implement written verification procedures (commonly called an “affirmative step”) that ensure the fish and fishery products they offer for entry into the U.S. were processed in accordance with the HACCP regulation.

The following are among USFDA’s key new import authorities and mandates under FSMA. Specific implementation dates specified in the law are noted in parentheses:

- Third Party Certification: FSMA establishes a program through which qualified third parties who implement certification programs that have been recognized by USFDA can certify that foreign food facilities comply with U.S. food safety standards. This certification may be used to facilitate the entry of imports.
- Certification for high risk foods: USFDA has the authority to require that high-risk imported foods be accompanied by a credible third party certification or other assurance of compliance as a condition of entry into the U.S.
- Voluntary qualified importer program: USFDA must establish a voluntary program for importers that provides for expedited review and entry of foods from participating importers. Eligibility is limited to, among other things, importers offering food from certified facilities.
- Authority to deny entry: USFDA can refuse entry into the U.S. of food from a foreign facility if USFDA is denied access by the facility or the country in which the facility is located.
- FSMA requires USFDA to develop a comprehensive plan to expand technical, scientific and regulatory food safety capacity of foreign governments and their respective food industries in countries from which foods are exported to the United States. Further, USFDA is required to develop the capacity-building plan in consultation with certain stakeholders, including representatives of the food industry, officials from other federal agencies, foreign government officials, non-governmental organizations (NGOs) that represent the interests of consumers, and other stakeholders. The capacity-building plan includes, as appropriate:
  1. recommendations for bilateral and multilateral arrangements and agreements, including providing for responsibilities of exporting countries to ensure food safety;
  2. provisions for secure electronic data sharing;
  3. provisions for mutual recognition of inspection reports;
  4. training of foreign governments and food producers on U.S. requirements for safe food;
  5. recommendations on whether and how to harmonize requirements under the Codex Alimentarius; and
  6. provisions for multilateral acceptance of laboratory methods and testing and detection techniques.

## 3. Conclusions

Oversight of the safety of the U.S. food supply continues to be a top priority for USFDA and USFDA has a strong regulatory program in place to ensure the safety of seafood products sold in the U.S. The corner stone of this program is the mandatory seafood HACCP regulation but also includes conducting foreign manufacturer inspections, conducting inspections of importers, collecting surveillance samples of imported goods at the time of entry, and working with our global partners. USFDA prioritizes these import-related activities based on risk.

USFDA will continue to work with our international partners to ensure the safety of imported seafood.
